# Molecular characterization of carcinosarcomas arising in the uterus and ovaries

**DOI:** 10.18632/oncotarget.26942

**Published:** 2019-06-04

**Authors:** Marta Brunetti, Antonio Agostini, Julie Staurseth, Ben Davidson, Sverre Heim, Francesca Micci

**Affiliations:** ^1^ Section for Cancer Cytogenetics, Institute for Cancer Genetics and Informatics, The Norwegian Radium Hospital, Oslo University Hospital, Oslo, Norway; ^2^ Department of Pathology, The Norwegian Radium Hospital, Oslo University Hospital, Oslo, Norway; ^3^ Institute of Clinical Medicine, Faculty of Medicine, University of Oslo, Oslo, Norway

**Keywords:** uterine carcinosarcomas, ovarian carcinosarcomas, mutational analysis, gene expression, miRNA expression

## Abstract

Gynaecological carcinosarcomas are rare biphasic tumours which are highly aggressive. We performed molecular investigations on a series of such tumours arising in the uterus (*n* = 16) and ovaries (*n* = 10) to gain more information on their mutational landscapes and the expression status of the genes *HMGA1/2*, *FHIT*, *LIN28A*, and *MTA1*, the pseudogenes *HMGA1P6* and *HMGA1P7*, and the miRNAs known to influence expression of the above-mentioned genes. In uterine carcinosarcomas (UCS), we identified mutations in *KRAS*, *PIK3CA*, and *TP53* with a frequency of 6%, 31%, and 75%, respectively, whereas in ovarian carcinosarcomas (OCS), *TP53* was the only mutated gene found (30%). An inverse correlation was observed between overexpression of *HMGA1/2*, *LIN28A*, and *MTA1* and downregulation of miRNAs such as let-7a, let-7d, miR26a, miR16, miR214, and miR30c in both UCS and OCS. *HMGA2* was expressed in its full length in 14 UCS and 9 OCS; in the remaining tumours, it was expressed in its truncated form. Because *FHIT* was normally expressed while miR30c was downregulated, not both downregulated as is the case in several other carcinomas, alterations of the epithelial-mesenchymal transition through an as yet unknown mechanism seems to be a feature of carcinosarcomas.

## INTRODUCTION

Carcinosarcomas (CS) of the female genital tract are rare but very aggressive biphasic neoplasms composed of a mixture of carcinomatous (malignant epithelial) and sarcomatous (malignant mesenchymal) components [1]. CS can arise in different organs of the female reproductive tract but are mostly seen in the uterus, where they account for less than 3% of all uterine malignancies [2, 3], and in the ovaries, where they account for 5% of ovarian cancers [4].

Uterine carcinosarcomas (UCS) and ovarian carcinosarcomas (OCS) are usually diagnosed in postmenopausal women at a median age of 65 years, frequently are at advanced stage when detected, and carry a poor prognosis [3]. 5-year survival rates have been reported at 50% at the early stages but only 10% for stage IV CS [5, 6].

Data on molecular genetic alterations, gene expression status, and epigenetic profiles of UCS and OCS are scarce and the few studies reported are based on small numbers of tumours [7–9]. Mutations of the tumour protein gene (*TP53*) are assumed to be the most frequent alteration, observed in 50% of analysed tumours [7, 10, 11]. Other mutations, reported at lower frequencies, affect the phosphatidylinositol-4,5-bisphosphate 3-kinase catalytic subunit alpha gene (*PI3K3CA*), the ki-ras2 kirsten rat sarcoma viral oncogene homolog (*KRAS*), the catenin beta 1 gene (*CTNNB1*), and the neuroblastoma RAS viral (V-Ras) oncogene homolog (*NRAS*) gene [7, 8, 12].

Dysregulation of chromatin remodelling genes has been shown in CS indicating their importance in CS tumourigenesis [7]. In UCS, the genes involved in chromatin modification include those encoding AT-rich interactive domain-containing proteins (*ARID1A* and *ARID1B*), histone methyltransferase mixed-lineage leukaemia protein 3 (*MLL3*), histone deacetylase modifier speckle-type POZ (*SPOP)*, and chromatin assembly factor bromodomain adjacent to zinc finger domain 1A (*BAZ1A*), all of which are mutated at frequencies varying from 18% to 36% [7]. Moreover, genes involved in chromosome dynamics were also found mutated, including those encoding DNA binding proteins, BCL6 corepressor (*BCOR*) and CCCTC-binding factor (*CTCF*), histone acetyl transferase E1A binding protein P300 (*EP300*), epigenetic activator zinc finger homeobox 3 (*ZFHX3*), and the nucleosome remodeling chromo domain helicase DNA binding protein 4 (*CHD4*) [12]. Some of these genes, including *BCOR* and *CHD4*, have been identified as mutated also in OCS [8].

Because both UCS and OCS may carry mutations in the histone genes *H2* and *H3*, mutations that may facilitate epithelial-mesenchymal transition (EMT), this has been proposed to lie at the heart of their role in sarcomatous transformation [8, 9]. However, since the genetic basis of these tumours still remains largely unexplored, we performed molecular genetic investigations hoping to gain more knowledge about the pathogenesis of this type of cancer.

To this aim we checked the mutation status of the isocitrate dehydrogenase 1 and 2 genes (*IDH1* and *IDH2*), telomerase reverse transcriptase (*TERT*) gene, the proto oncogenes *BRAF*, *HRAS*, *KRAS*, and *NRAS*, the histone *H3F3A*, *CTNNB1*, and *PIK3CA*, and *TP53* in a series of CS arising in the uterus and ovaries. We also investigated the methylation status of the promoter of O6-methylguanine-DNA methyltransferase gene (*MGMT*).

To obtain more insight into the role of chromatin regulation genes and their pathways, we analysed the expression status of the high mobility group AT-Hook genes (*HMGA1* and *HMGA2*), the pseudogenes *HMGA1P6* and *HMGA1P7*, and the fragile histidine triad (*FHIT*), lin-28 homolog A (*LIN28A*) and metastasis associated 1 (*MTA1*) genes, as well as these genes’ possible regulation by miRNAs such as let-7a, let-7d, miR26a, miR16, miR214, and miR30c.

## RESULTS

### Mutation and methylation analyses

**Figure 1:**
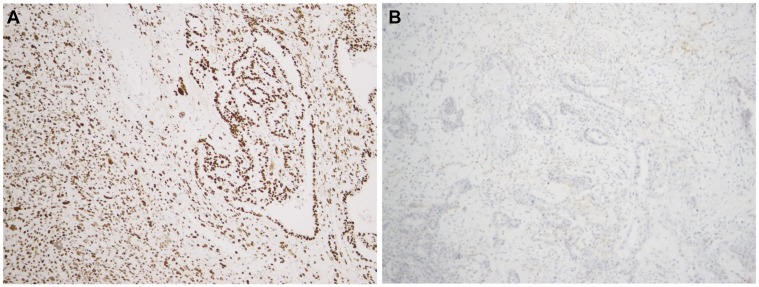
p53 immunostaining in two uterine carcinosarcomas showing the two aberrant patterns, i.e. diffuse strong expression and entirely negative expression in panels (**A** and **B**), respectively.

All tumours analysed for *IDH1*, *IDH2*, *TERT*, *CTNNB1*, *BRAF*, *H3F3A*, *KRAS*, *HRAS*, *NRAS*, *PIK3CA*, and *TP53* mutation status gave informative results. Whereas no tumour showed a mutated sequence for *IDH1*, *IDH2*, *TERT*, *BRAF*, *H3F3A*, *HRAS,* *NRAS* or *CTNNB*, a few were found to be mutated in *KRAS*, *PIK3CA*, and/or *TP53*. An overview of the findings is shown in Table 1. We identified a c.175G>A *KRAS* mutation in one of 16 UCS (case 8; Table 1). *PIK3CA* mutations were found in five of 16 UCS but in none of the OCS. More specifically, a c.3073A>G mutation was detected in case 8, a c.1637A>G in case 9, a c.3140A>G in cases 11 and 16, and a c.1634A>G in case 12 (Table 1). *TP53* was found mutated in 12 of 16 UCS (cases 1, 2, 3, 4, 5, 6, 8, 9, 10, 11, 16, and 17; 75% of the uterine CS) and in three of ten OCS (cases 18, 19, and 22; 30%). Details about the *TP53* mutations are listed in Table 1. The expression of aberrant TP53 was confirmed by immunohistochemistry (Figure 1).

**Table 1:** Mutation status of *KRAS*, *CTNNB1*, *PIK3CA*, and *TP53* and TP53 protein expression

Case/lab no	Diagnosis	*KRAS*	*CTNNB1*	*PIK3CA*	*TP53*	TP53 carcinoma	TP53 sarcoma
1/03–113	UCS	-	-	-	c.722C>T	aberrant +	aberrant +
2/03–221	UCS	-	-	-	c.383_388delCTGCCC	WT	aberrant +
3/08–1637	UCS	-	-	-	rs28934578 (ARG175HIS)	aberrant +	aberrant +
4/03–684	UCS	-	-	-	rs28934578 (ARG175HIS)	aberrant +	aberrant +
5/03–1023	UCS	-	-	-	c.722C>A	aberrant +	aberrant +
6/08–521	UCS	-	-	-	c.818G>A	aberrant +	missing
7/05–1309	UCS	-	-	-	-		
8/0992–160	UCS	c.175G>A	-	c.3073A>G	c.817C>T		
9/1002–102	UCS	-	-	c.1637A>G	c.844C>G	aberrant +	aberrant +
10/1002–186	UCS	-	-	-	c.794T>C	aberrant +	aberrant +
11/00–701	UCS^*^	-	-	c.3140A>G	c.817C>T	aberrant +	aberrant +
12/02–819	UCS^*^	-	-	c.1634A>G	-	WT	WT
13/06–539	OCS	-	-	-	-	aberrant –	aberrant –
14/1002–356	UCS	-	-	-	-		
15/02–873	UCS	-	-	-	-	aberrant +	aberrant +
16/01–73	UCS	-	-	c.3140A>G	c.215C>G		
17/06–1577	UCS	-	-	-	c.558T>A	aberrant -	aberrant +
18/08–974	OCS	-	-	-	c.503A>C	aberrant +	aberrant +
19/009–90	OCS	-	-	-	c.815T>G	aberrant +	aberrant –
20/01–139	OCS	-	-	-	-	aberrant +	aberrant +
21/008–35	OCS	-	-	-	-	WT	WT
22/0992–0288	OCS	-	-	-	c.393_395delCAA	aberrant +	aberrant +

^*^UCS previously investigated in Micci *et al*., 2004

No *MGMT* promoter methylation was detected in the present series, suggesting that the gene is not involved in CS tumourigenesis.

### Expression analyses

**Figure 2:**
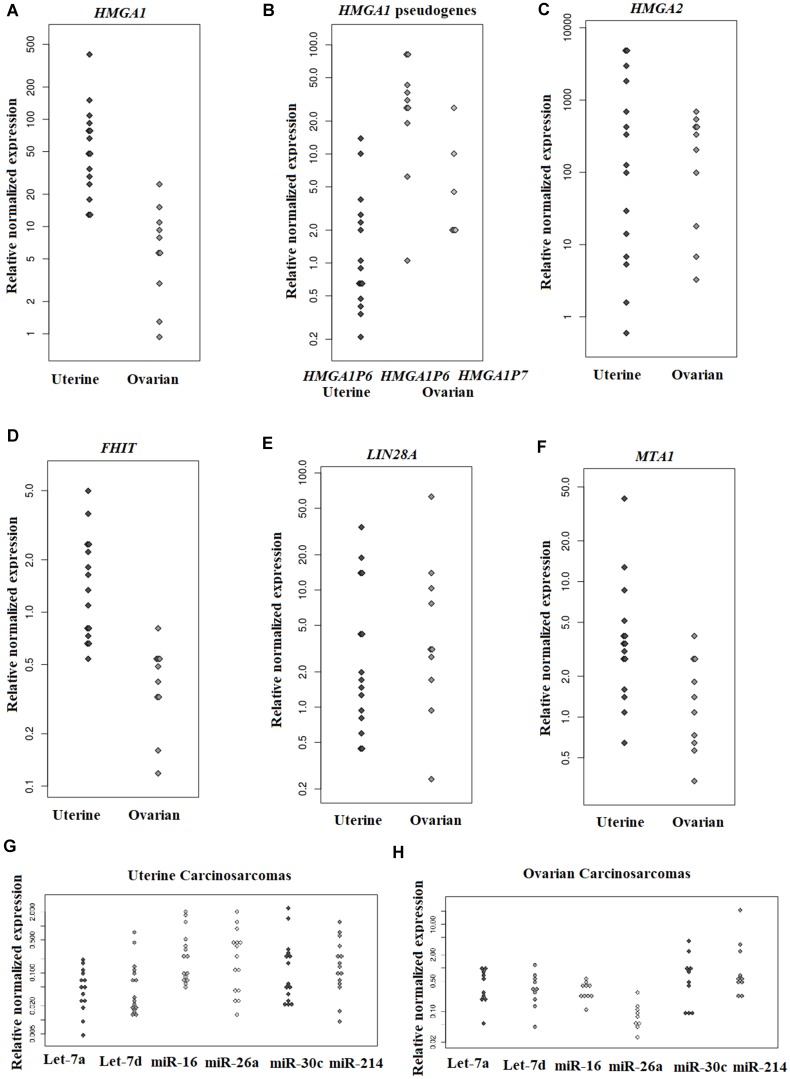
Genes and miRNA expression levels in uterine and ovarian carcinosarcomas assessed by Real-Time PCR. The relative expression of *HMGA1* (**A**), *HMGA1* pseudogenes (**B**), *HMGA2* (**C**), *FHIT* (**D**), *LIN28A* (**E**), *MTA1* (**F**) in uterine and ovarian CS; let-7a, let-d, miR16, miR-26a, miR-30c, and miR-214 in UCS (**G**) and in OCS (**H**).

An overview of the expression status for the genes and miRNAs investigated is given in Tables 2 and 3. *HMGA1* was found expressed in UCS and OCS (Figure 2A). *HMGA1P6* was expressed in seven of 15 UCS and, at high levels, in all OCS (Figure 2B). *HMGA1P7* was not expressed in UCS but was expressed in six of ten OCS (Figure 2B). *HMGA2* was expressed at high levels in both uterine and ovarian CS (Figure 2C). *FHIT* was found normally expressed in both UCS and OCS (Figure 2D). *LIN28A* was found upregulated in six of 15 UCS and in most OCS (nine of ten) (Figure 2E). *MTA1* was found overexpressed in UCS, whereas no substantial overexpression was identified in OCS (Figure 2F).

**Table 2:** Overview of the expression status of genes and miRNAs investigated in the CS

Case/lab no	Histology	*HMGA1*	*HMGA2*	*FHIT*	*LIN28A*	*HMGA1P6*	*HMGA1P7*	*MTA1*	Let-7a	Let-7d	miR26a	miR16	miR214	miR30c
1/03–113	UCS	↑	↑	↑	↑	↑	N/A	↑	↓	↓	-	-	↓	↓
2/03–221	UCS	↑	↑	↑	-	-	N/A	↑	↓	↓	↓	↓	-	↓
3/08–1637	UCS	↑	↑	↑	↑	-	N/A	↑	↓	↓	↓	↓	↓	↓
4/03–684	UCS	↑	↑	↑	-	-	N/A	↑	↓	↓	↓	↓	↓	↓
5/03–1023	UCS	↑	↑	↑	↑	↑	N/A	↑	↓	↓	-	↓	↓	↓
6/08–521	UCS	↑	↑	↑	-	↑	N/A	↑	↓	↓	↓	↓	↓	↓
7/05–1309	UCS	↑	↑	↑	↑	-	N/A	↑	↓	↓	↓	↓	↓	↓
8/0992–0160	UCS	↑	↑	↑	-	-	N/A	↑	↓	↓	↓	↓	↓	↓
9/1002–0102	UCS	↑	↑	↑	-	-	N/A	↑	↓	↓	↓	↓	↓	↓
10/1002–186	UCS	↑	↑	↑	-	↑	N/A	↑	↓	↓	↓	↓	↓	↓
11/00–701	UCS^*^	↑	↑	↑	-	-	N/A	↑	↓	↓	↓	↓	↓	↓
12/02–819	UCS^*^	↑	↑	↑	↑	-	N/A	↑	↓	↓	↓	↓	↓	↓
13/06–539	OCS	↑	↑	↓	↑	↑	N/A	↓	↓	↓	↓	↓	↓	-
17/06–1577	UCS	↑	↑	↓	↑	↑	↑	↓	↓	↓	↓	↓	↑	-
18/08–974	OCS	↑	↑	↓	↑	↑	↑	↓	↓	↓	↓	↓	↑	-
19/09–90	OCS	↑	↑	↓	-	↑	↑	↓	↓	↓	↓	↓	↓	-
20/01–139	OCS	↑	↑	↓	-	↑	N/A	↓	↓	↓	↓	↓	↓	↑
21/08–35	OCS	↑	↑	↓	↑	↑	↑	↓	↓	↓	↓	↓	↓	-
22/0992–0288	OCS	↑	↑	↓	↑	↑	↑	↓	↓	↓	↓	↓	↓	↑
23/03–568	UCS	↑	↑	↑	↑	↑	N/A	↑	↓	↓	↓	↓	↓	↓
24/01–104	UCS^*^	↑	↑	↑	-	↑	N/A	↑	↓	↓	-	-	↓	↓
25/01–1056	UCS^*^	↑	↑	↑	-	↑	N/A	↑	↓	↓	↓	-	↓	↓
26/05–268	OCS	↑	↑	↓	↑	-	↑	↓	↓	↓	↓	↓	↓	↑
27/05–1076	OCS	↑	↑	↓	↑	↑	N/A	↓	↓	↓	↓	↓	↓	↑
28/02–1150	OCS	↑	↑	↓	↑	↑	N/A	↓	↓	↓	↓	↓	↓	↑

^*^UCS previously investigated in Micci *et al*., 2004

The miRNAs let-7a, let-7d, miR-16, miR26a, and miR-30c were found downregulated in both UCS and OCS. miR-214 was downregulated in all UCS, whereas it was upregulated in three out of ten ovarian tumours but downregulated in the remaining seven (Table 3; Figures 2G and 2H). The Mann-Whitney *U* Test for statistical analysis was used to compare uterine and ovarian carcinosarcomas for gene and miRNA expression. No significantly different expression between the two tumour types (*p >* 0.05) was seen for any of the genes or miRNAs examined.

**Table 3:** Mean and median of genes and miRNA expression

Gene	UCS		OCS	
	Mean	Median	Mean	Median
*HMGA1*	81.3	47.1	8.5	7.8
*HMGA1P6*	2.6	0.9	39.7	32.4
*HMGA1P7*			5.1	2.0
*HMGA2*	1146.2	117.7	279.2	310.2
*FHIT*	1.7	1.2	0.4	0.4
*LIN28A*	1.7	1.2	12.3	3.3
*MTA*1	6.4	3.3	1.7	1.3
**miRNA**				
let-7a	0.06	0.04	0.5	0.6
let-7d	0.12	0.03	0.4	0.3
miR-16	0.4	0.2	0.3	0.3
miR26a	0.4	0.21	0.09	0.07
miR-30c	0.3	0.05	1.16	0.1
miR-214	0.2	0.1	3.5	0.6

**Figure 3:**
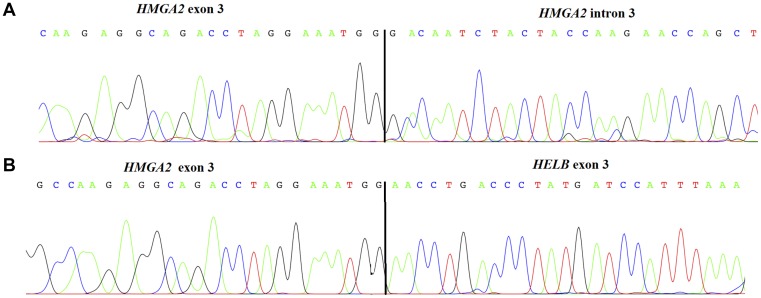
Chromatogram and sequence of *HMGA2* truncated transcript found in an uterine carcinosarcoma (case 4) showing the junction between exon 3 and the intronic region (**A**). Chromatogram of *HMGA2* truncated transcript found in a uterine carcinosarcomas (case 25) showing a fusion between *HMGA2* and *HELB* (**B**).

We performed 3′ RACE-PCR on three tumours (cases 4, 18, and 25) that lacked 3′ sequences. In case 4, an UCS, exon 3 of *HMGA2* was fused with part of the third intron, 78 kb downstream from the exon 3/intron 3 splicing site (Figure 3A). Case 25, a UCS, showed an in-frame fusion between *HMGA2* (exon 3) and the Homo sapiens helicase (DNA) B (*HELB*; NM_033647; exon 3) located in the same chromosomal region (12q14.3) but 467 Kb distally (Figure 3B). Case 18, an OCS, did not give informative sequencing results.

## DISCUSSION

In the present study, mutations in *KRAS*, *PIK3CA*, and *TP53* were found in 6%, 31%, and 75% of UCS, respectively, in line with previous findings [7, 8, 10]; (COSMIC database https://cancer.sanger.ac.uk/cosmic). In OCS, *KRAS* and *PIK3CA* were not mutated, whereas 30% of OCS carried *TP53* mutations.

Genetic alterations of *TP53* have been thoroughly investigated in human cancer [13]. It is known that *TP53* mutations occur during CS tumourigenesis, causing the gene to lose its tumour suppressive function, indicating its role as an early pathogenetic driver [8, 14]. The distribution pattern of *TP53* mutations found by us was in line with that found in previous studies [7]. Alterations in *TP53* were previously observed in most UCS and OCS analysed [8, 9]. The *TP53* mutations targeted the core of the DNA-binding domain, resulting in loss of its regulatory function on gene expression and accumulation of non-functional p53 protein. We validated p53 expression by immunohistochemistry, finding a correlation between *TP53* mutational status and p53 expression pattern. The latter analysis showed equal expression of the protein in both components (carcinomatous and sarcomatous) suggesting that there is no leading components for p53 expression as a “driving force” of tumourigenesis.

*HMGA1* and *HMGA2* are members of the high-mobility group AT-hook family and are involved in a variety of biological processes from chromosome dynamics to gene regulation [15]. They are usually expressed during embryonic development [15, 16] but not in adult normal tissues [17]. The genes were found overexpressed and/or targeted as part of the pathogenesis of many different tumours, both benign [18] and malignant [19], including mesenchymal [20] and epithelial [21] ones. HMGA proteins are involved in different pathogenic processes, but exert their main tumourigenic effect activating and sustaining epithelial-mesenchymal transition (EMT) [22]. We found *HMGA1* overexpressed in both UCS and OCS. Interestingly, *HMGA2* was expressed at higher levels than its homologue in UCS as well as in OCS. The mechanisms of regulation of these two genes are not fully understood, but non-coding RNA dysregulation and chromosomal alterations are the two main causes leading to upregulation of *HMGA1* and *HMGA2* in cancer [18, 20, 23, 24]. The *HMGA1*-targeting miRNAs let-7a [24], miR-26a [21], miR-16 [25], and miR-214 [26] were downregulated in CS of both sites in the present study, giving the impression that these cancers do not differ from other malignancies in this regard. The *HMGA1* pseudogenes *HMGA1P6* and *HMGA1P7* were found to be implicated in the downregulation of the aforementioned miRNAs [27] and the overexpression of *HMGA1*. The *HMGA1P6* and *HMGA1P7* pseudogenes conserve seed matches for the *HMGA1*-targeting miRNAs and operate as decoys for these miRNAs, contributing to *HMGA1* overexpression [28]. In UCS, only *HMGA1P6* was expressed, while both *HMGA1P6* and *HMGA1P7* were expressed at high levels in OCS. The findings suggest that these pseudogenes may contribute to *HMGA1* deregulation in gynaecological CS.

The mechanisms leading to expression of *HMGA2* are still partly obscure, but interaction between miRNAs and the *HMGA2* 3′untranslated region (3′UTR) seems to be crucial [29]. It has been shown that the *HMGA2* 3′UTR has many regulatory sequences which are targeted by different families of miRNAs [29], and it is thought that miRNA-dependent repression is the main mechanism controlling *HMGA2* expression [30–32]. We observed upregulation of *HMGA2* with miRNA downregulation in both UCS and OCS, providing another piece of evidence that the interaction between the two is important also in gynaecological CS. Another indication pointing in the same direction has been the identification of disrupted forms of *HMGA2*, due to rearrangements of chromosomal band 12q15 (the band where the gene is located), that are consistently seen in different benign mesenchymal tumours but also in some malignant neoplasms such as ovarian carcinomas and leukemia [20, 33–36]. These alterations involve exon 3 and cause deletion of downstream regions leading to a truncated transcript that can evade miRNA-dependent gene silencing. As we have seen a 3′ rearranged form of *HMGA2* in only two of 15 UCS and one of ten OCS, we hypothesize that mechanism(s) other than *HMGA2*-rearrangements may be active in these tumours.

The *HMGA2*-targeting miRNAs let-7a, let-7d, miR-30c, and miR-26a were found highly downregulated in all UCS examined. Only let-7a, let-7d, and miR-26a were downregulated in OCS, whereas miR-30c was normally expressed.

Allegedly, *LIN28A* causes downregulation of the let-7 family of miRNAs, inhibiting the maturation of both pri- and pre-let-7 [37]. The gene was found expressed in both UCS and OCS, suggesting possible involvement in the downregulation of let-7 miRNAs in CS generally.

Expression of *FHIT* and miR-30c has been shown to be inversely correlated with *HMGA2* expression in lung cancer [31] and squamous cell carcinoma of the vulva [38]. *FHIT* and miR-30c downregulation causes *HMGA2* upregulation promoting EMT [31, 38]. We did not find any similar correlation between *FHIT* and miR-30c in the CS analysed, as *FHIT* was normally expressed while miR30c was highly downregulated in UCS, whereas *FHIT* was downregulated while miR30c was normally expressed in OCS. We therefore suggest that other/additional mechanisms and/or genes are involved in the pathway leading to overexpression of *HMGA2* in this tumour type. More specifically, there could be other molecules than FHIT involved in miR30c downregulation.

*MTA1* has emerged as one of several highly deregulated oncogenes in human cancer, possibly because of its dual nature as corepressor and coactivator [39]. The MTA1 protein forms the NuRD chromatin remodeling complex and regulates expression of a wide range of genes involved in carcinogenesis such as *HIF*α [40] and *ER*α [41]. *MTA1* is regulated by miR-30c and miRNA downregulation is associated with *MTA1* upregulation in endometrial [42] and ovarian [43] cancer. In UCS, we found the same inverse correlation reported by others [42, 43] where *MTA1* is overexpressed and miR-30c downregulated, whereas the expression levels of miR-30c and *MTA1* in our series of OCS were generally normal.

In conclusion, our analyses showed that miRNAs responsible for *HMGA* expression are downregulated in CS of the female genital tract. The downregulation was more pronounced in UCS compared to OCS (the mean was 10-fold lower). This may explain the consistently higher levels of *HMGA1* and *HMGA2* in UCS compared to OCS. Future studies should be focused on seeing if mutations in the above-mentioned genes are present in both tumour components, i.e., the sarcomatous and carcinomatous areas, or only in one of them. Unfortunately, in our tumours these parts were so intermingled that it was not possible to separate them and run parallel tests.

## MATERIALS AND METHODS

### Tumour material

The material consisted of fresh samples from 16 UCS and ten OCS surgically removed at The Norwegian Radium Hospital between 2000 and 2010. Four of the uterine carcinosarcomas were previously karyotyped and tested by comparative genomic hybridization (CGH) for chromosomal aberrations and genomic imbalances [44]. For historical reasons and to facilitate relevant electronic searches, we refer to all tumours arising in the uterine adnexa as ovarian throughout the manuscript; this should not be interpreted as reflecting certainty that they arise from cells of the ovary and not from the fallopian tube. All samples had a minimum of 50% of tumor cell content, the majority >80%; no difference was noted between uterine and ovarian tumors. The study was approved by the Regional Committee for Medical and Health Research Ethics, South-East Norway (REK Sør-Øst; http://helseforskning.etikkom.no).

### DNA and RNA extraction and cDNA synthesis

DNA extraction was performed using the Maxwell 16 extractor (Promega, Madison, WI, USA) and Maxwell 16 Tissue DNA Purification kit (Promega) according to the manufacturer’s recommendations. RNA extraction was performed using the miRNeasy kit (Qiagen, Hilden, Germany) and QIAcube (Qiagen). The concentration was measured with QIAxel (Qiagen). One microgram of extracted RNA was reverse-transcribed in a 20 μL reaction volume using the iScript Advanced cDNA Synthesis kit according to the manufacturer’s instructions (Bio-Rad Laboratories, Oslo, Norway).

### Mutational and methylation analyses

Mutational analyses of *IDH1*, *IDH2*, *TERT*, *CTNNB1*, *BRAF*, *H3F3A*, and *TP53* were performed according to previously described protocols [45, 46]. Primers for *HRAS*, *KRAS*, *NRAS*, and *PIK3CA* are listed in Table 4. The mutational analyses were performed using M13-linked PCR primers designed to flank and amplify targeted sequences. The thermal cycling for *HRAS* and *NRAS* included an initial step at 95° C for 10 min followed by 35 cycles at 96° C for 3 sec, 58° C for 15 sec, 30 sec at 68° C, and a final step at 72° C for 2 min. The thermal cycling for *KRAS* was set to 94° C for 30 sec followed by 35 cycles of 7 sec at 98° C, 30 sec at 54° C, 1 min at 77° C, and a final step at 68° C for 5 min. The thermal cycling for *PIK3CA* was set to 95° C for 10 min followed by 35 cycles of 3 sec at 96° C, 15 sec at 62° C, 30 sec at 68° C, and a final step at 72° C for 2 min. Direct sequencing was performed using a 3500 Genetic Analyzer (Applied Biosystems). The BLAST (https://blast.ncbi.nlm.nih.gov/Blast.cgi) and BLAT (https://genome-euro.ucsc.edu/cgi-bin/hgBlat) programs were used for computer analysis of sequence data.

**Table 4:** Primers used for molecular investigations

Primer name	Sequence	Position	Gene	Accession number
**Mutational analyses**				
HRAS-EXON2FW	5′-CATTAAGAGCAAGTGGGGGCG-3′	5973–5993	*HRAS*	NG_007666.1
HRAS-EXON2REV	5′-CGAGGGACTCCCCTCCTCTA-3′	6466–6485	*HRAS*	NG_007666.1
HRAS-EXON3FW	5′-AGGGGCATGAGAGGTACCAG-3′	6516–6535	*HRAS*	NG_007666.1
HRAS-EXON3REV	5′-CATCCAGGACATGCGCAGA-3′	6871–6889	*HRAS*	NG_007666.1
KRAS-EXON2FW	5′-AAGGTACTGGTGGAGTATTTG-3′	10439–10459	*KRAS*	NG_007524.1
KRAS-EXON2REV	5′-ATGAAAATGGTCAGAGAAACC-3′	10707–10727	KRAS	NG_007524.1
KRAS-EXON3FW	5′-TTGAAGTAAAAGGTGCACTG-3′	28457–28475	KRAS	NG_007524.1
KRAS-EXON3REV	5′-AATTACTCCTTAATGTCAGCTT-3′	28710–28731	KRAS	NG_007524.1
NRAS EX 2 FW	5′-GGCTCGCCAATTAACCCTGA-3′	5681–5700	NRAS	NG_007572.1
NRAS EX 2 REV	5′-TCCGACAAGTGAGAGACAGGA-3′	5876–5886	*NRAS*	NG_007572.1
NRAS EX 3 FW	5′-GCATTGCATTCCCTGTGGTTT-3′	7841–7871	*NRAS*	NG_007572.1
NRAS EX 3 REV	5′-GTGTGGTAACCTCATTTCCCCA-3′	8150–8171	NRAS	NG_007572.1
PIK3CA- Ex10F1	5′-ATCATCTGTGAATCCAGAGGGGAA-3′	74619–74642	PIK3CA	NG_027450.2
PIK3CA- Ex10R1	5′- CATGCTGAGATCAGCCAAATTCAG-3′	74868–74891	PIK3CA	NG_012113.2
PIK3CA- Ex21F1	5′-CATCATTTGCTCCAAACTGACCAA-3′	90528–90551	PIK3CA	NG_012113.2
PIK3CA- Ex21R1	5′-TCATGGATTGTGCAATTCCTATGC-3′	90922–90945	PIK3CA	NG_012113.2
**Expression analyses**				
HMGA2-846F1	5′ –CCACTTCAGCCCAGGGACAACCT- 3′	846–868	HMGA2	NM_003483.4
HMGA2-1021R1	5′ -CCTCTTGGCCGTTTTTCTCCAGTG- 3′	1021–1044	HMGA2	NM_003483.4
HMGA2-1112R1	5′ –CCTCTTCGGCAGACTCTTGTGAGGA3′	1112–1136	HMGA2	NM_003483.4
HMGA2F1	5′-TCAGAAGAGAGGACGCGG-3′	883–900	HMGA2	NM_003483.4
HELBR1	5′-CTTCAAATCAGTCATTCTTTGGGT- 3′	66306281–66306304^*^	HELB	NM_033647.4
HMGA2F4	5′ -AAAAACAAGAGTCCCTCTAAAGCA- 3′	977–1000	HMGA2	NM_003483.4
HELBR4	5′-TTGCAGTTTCCGAAGATAATGGA- 3′	693–715	HELB	NM_033647.4

^*^Genomic coordinates ch 12 GRch38p7 primary assembly

Methylation-specific quantitative polymerase chain reaction (MSP-qPCR) analysis of the *MGMT* promoter was performed as reported earlier [45].

### Real-Time polymerase chain reaction (Real-Time PCR)

Expression level of the selected genes and miRNAs was assessed by Real-Time PCR using the CFX96 Touch Real-Time detection system (Bio-Rad Laboratories, Oslo, Norway). The reactions were carried out in triplicate using the TaqMan Universal Master Mix II with UNG (Applied Biosystems, Foster City, CA, USA) following the manufacturer’s protocol. Human Universe Reference Total RNA (Clontech, Mountain View, CA, USA) was used as internal reaction control. The Human Ovary Total RNA (MVP Total RNA Human Ovary, Agilent Technologies, Santa Clara, CA, USA) and one sample of normal uterus tissue were used as reference for relative expression normalization. Two stably expressed known genes (housekeeping genes) were used as references as these were previously evaluated as stable in gynaecological tumours [47]. The Real-Time data were analysed with Bio-Rad CFX manager 3.1 (Bio-Rad). The normalized expression was calculated using the 2^-ΔΔCt^ (Livak) method [48].

One μg of extracted total RNA was reverse-transcribed in a 20 μL reaction volume using iScript Advanced cDNA Synthesis Kit according to the manufacturer’s instructions (Bio-Rad Laboratories, Oslo, Norway). Gene expression was assessed with Real-Time PCR using the TaqMan Gene Expression Assays (Applied Biosystems) for the following genes: *HMGA1* (Hs_00852949_g1), *HMGA2* (Hs_04397751_m1), FHIT (Hs_00179987_m1), *LIN28A* (Hs_00702808_Gh), *HMGA1P6* (ARYMJHZ), and *HMGA1P7* (Hs04232395_m1). The *UBC* (Hs01871556_m1) and *TBP* (Hs00427620_m1) genes were used as references.

Ten ng of total RNA were reverse transcribed using the TaqMan microRNA Reverse Transcription Kit (Applied Biosystems) following the manufacturer’s protocol. miRNA expression was assessed with Real-Time PCR using the TaqMan microRNA assays (Applied Biosystems) for let-7a (RT: 000377), let-7d (RT: 002283), miR-26a (RT: 000405), miR-16 (RT: 000391), miR-214 (TM: 002306), and miR30c (TM:000419). The *RNU6B* gene (TM:001093) was used as a reference as it was previously validated as stable in different gynaecological tumours [38, 49].

### Reverse transcriptase-polymerase chain reaction (RT-PCR)

cDNA equivalent to 10 ng RNA was amplified using the Takara Premix Ex Taq (Takara-Bio, Europe/SAS, Saint-Germain-en-Laye, France). The primers used for PCR reactions are listed in Table 4. The primer combination HMGA2-846F1 and HMGA2-1021R1 was used to amplify the region between exons 1 and 3, whereas the primer combination HMGA2-846F1 and HMGA2-1112R1 was used for exons 1 to 5 (Table 4). The PCR cycling program was previously reported [35].

### 3′ Rapid amplification of cDNA ends – PCR (3′ RACE–PCR)

For 3′-RACE-PCR, 100 ng of total RNA were reverse-transcribed in a 20 μL reaction volume using a previously described protocol [45]. To validate the fusion between *HMGA2* (exon 3) and *HELB* (exon 3), RT-PCR was performed with specific primer combinations for the two genes. The PCR cycling program was: 30 sec at 94° C followed by 35 cycles of 7 sec at 98° C and 1 min at 55° C, 1 min at 72° C, and a final step at 72° C for 2 min.

### Immunohistochemistry

Formalin-fixed, paraffin-embedded sections were analysed for p53 protein expression in 19 tumours from which material was available using the Dako EnVision™ Flex+ System (K8012; Dako, Glostrup, Denmark). Epitope unmasking was carried out in a high pH solution. Sections were incubated with a 0.3% hydrogen peroxide (H_2_O_2_) solution for 5 min to block endogenous tissue peroxidase activity. Sections were then incubated with a mouse monoclonal p53 primary antibody (clone DO-1, catalogue #sc-126, Santa Cruz Biotechnology, Santa Cruz CA, USA) and treated with EnVision™ Flex+ mouse linker (15 min) and EnVision™ Flex/HRP enzyme (30 min), stained for 10 min with 3`3 diaminobenzidine tetrahydrochloride (DAB), counterstained with haematoxylin, dehydrated, and mounted in Richard-Allan Scientific Cyto seal XYL (Thermo Fisher Scientific, Waltham, MA, USA). Positive control consisted of colon carcinoma.
